# Electromyographic features and efficacy of orofacial myofunctional treatment for skeletal anterior open bite in adolescents: an exploratory study

**DOI:** 10.1186/s12903-021-01605-0

**Published:** 2021-05-07

**Authors:** Hong Hong, Yue Zeng, Xiaomin Chen, Caixia Peng, Jianqing Deng, Xueqin Zhang, Lidi Deng, Yongjian Xie, Liping Wu

**Affiliations:** 1 Hospital of Stomatology, Guanghua School of Stomatology, Guangdong Provincial Key Laboratory of Stomatology, Sun Yat-Sen University, Guangzhou, 510055 People’s Republic of China; 2 Guangzhou Panyu Central Hospital, Guangzhou, 511400 People’s Republic of China; 3 Department of Stomatology, Baoan Maternal and Child Health Hospital, Jinan University, Shenzhen, 518106 People’s Republic of China; 4 The Eighth Affiliated Hospital, Sun Yat-Sen University, Shenzhen, 518000 People’s Republic of China; 5 Department of Periodontology, Peking University School and Hospital of Stomatology, National Engineering Laboratory for Digital and Material Technology of Stomatology, Beijing Key Laboratory of Digital Stomatology, Beijing, 100081 People’s Republic of China

**Keywords:** Skeletal anterior open bite, Orofacial myofunctional status, Orofacial myofunctional therapy

## Abstract

**Background:**

Due to the multifactorial aetiology and unpredictable long-term stability, skeletal anterior open bite (SAOB) is one of the most intractable conditions for orthodontists. The abnormal orofacial myofunctional status (OMS) may be a major risk factor contributing to the development and relapse of SAOB. This study is aimed at evaluating the OMS and the efficacy of orofacial myofunctional therapy (OMT) alone for SAOB subjects.

**Methods:**

Eighteen adolescents with SAOB (4 males, 14 females; age: 12–18 years) and eighteen adolescents with normal occlusion (2 males, 16 females; age: 12–18 years) were selected. The electromyographic activity (EMGA) associated with mastication and closed mouth state was measured. Lateral cephalography was used to evaluate craniofacial morphology. Wilcoxon signed rank tests and t-tests were performed to evaluate myofunctional and morphological differences. Pearson or Spearman correlation analysis was used to investigate the correlations between EMGA and morphological characteristics. SAOB subjects were given OMT for 3 months, and the EMGA was compared between before and after OMT.

**Results:**

During rest, anterior temporalis activity (TAA) and mentalis muscle activity (MEA) increased in SAOB subjects, but TAA and masseter muscle activity (MMA) decreased in the intercuspal position (ICP); and upper orbicularis activity (UOA) and MEA significantly increased during lip sealing and swallowing (*P* < 0.05). Morphological evaluation revealed increases in the FMA, GoGn-SN, ANS-Me, N-Me, L1-MP, U6-PP, and L6-MP and decreases in the angle of the axis of the upper and lower central incisors and OB in SAOB subjects (*P* < 0.05). TAA, MMA and anterior digastric activity (DAA) in the ICP were negatively correlated with vertical height and positively correlated to incisor protrusion. MEA was positively correlated with vertical height and negatively correlated with incisor protrusion; and the UOA showed a similar correlation in ICP, during sealing lip and swallowing. After SAOB subjects received OMT, MEA during rest and TAA, MMA and DAA in the ICP increased, while UOA and MEA decreased (*P* < 0.05).

**Conclusion:**

SAOB subjects showed abnormal OMS features including aberrant swallowing patterns and weak masticatory muscles, which were interrelated with the craniofacial dysmorphology features including a greater anterior facial height and incisor protrusion. Furthermore, OMT contributes to OMS harmonization, indicating its therapeutic prospect in SAOB.

## Background

Anterior open bite malocclusion is defined as the lack of vertical overlap or contact between the upper and lower incisors with occlusion of the posterior teeth. It can be classified as dentoalveolar or skeletal malocclusion. Certain morphological features, such as increased gonial and steep occlusal plane angles and increased anterior facial height, are usually present in individuals with skeletal anterior open bite (SAOB) [1–4]. Due to the multifactorial aetiology and unpredictable long-term stability of SAOB, it is considered one of the most intractable conditions to treat by many orthodontists.

Recent research has demonstrated the high prevalence of harmful oral habits, such as thumb or dummy sucking, mouth breathing, and tongue-thrust swallowing, among children with open bite [5–8], mainly ranging in age from 0 to 3 years old. One school of thought believes that, druing the long process of oromaxillofacial muscle development, harmful oral habits contribute to the development of an abnormal orofacial myofunctional status (OMS) [9, 10]. Several cross-sectional studies found that the abnormal OMS, in which inappropriate and uneven muscular pressure on facial bones can influence directional growth over time and eventually result in open bite [11–13]. Furthermore, a ten-year follow-up research showed that the dysfunctional neuromuscular pattern formed in long-term development could also lead to unfavourable results and relapse after treatment [14]. These facts suggest that the abnormal OMS of SAOB is a potential major risk factor that contributes to the development, treatment and relapse of open bite. Others support the opinion that the existence of harmful oral habits in the developing craniofacial structures is the consequence of the existing malocclusion of open bite, which is the form that determines OMS [15, 16].

Orofacial myofunctional therapy (OMT), which is often considered as an adjunct to conventional orthodontic treatment, has been shown to be effective in harmonizing the OMS based on its improvement of musculature and orofacial function. Degan VV [17] highlighted that myofunctional therapy associated with the removal of sucking habits contributed to a better and faster amelioration of the swallowing pattern and the tongue rest position. Korbmacher [18] demonstrated that an appliance-based orofacial muscle training protocol exhibited 65% effectiveness in altering habitual mouth breathing to nasal breathing. However, a recent systematic review and meta-analysis found that although myofunctional treatment in the deciduous and mixed dentition children appeared to be promising to correct anterior open bite, the quality of the existing evidence was questionable [19]. Moreover, inadequate attention has been paid to the correlation between the OMS and morphological features in SAOB and the potential benefits of OMT regarding the myofunctional status and orthodontic treatment.

In the present study, we compared the EMGA of different muscles under four conditions between adolescents with SAOB or normal occlusion, and we performed an analysis of correlations between the EMGA and morphological features of adolescents with SAOB. In addition, we compared the myofunctional status of oromaxillofacial muscles in SAOB subjects before and after receiving OMT. The aim of this study was to obtain a better understanding of the OMS and its relationship with craniofacial dysmorphology in SAOB subjects and to propose preliminary treatment strategies targeting early intervention, thereby facilitating orthodontic treatment.

## Methods

### Subjects

Eighteen adolescents with SAOB (4 males, 14 females; age: 12–18 years) and eighteen adolescents with normal occlusion (2 males, 16 females; age: 12–18 years) were selected for comparison of the OMS. Three female adolescents with SAOB were lost to follow-up during OMT, so a total of fifteen adolescents with SAOB (4 males, 11 females; age: 12–18 years) finished the OMT regimen. All of the subjects were diagnosed at the orthodontic department of the Hospital of Stomatology, Sun Yat-sen University. This study was performed in accordance with the Declaration of Helsinki, and the Medical Ethics Committee of the Hospital of Stomatology, Sun Yat-sen University, approved the study (Protocol Title: The Electromyographic Activity Of Oromaxillofacial Muscles In Adolescents With Skeletal Anterior Open Bite, No. KQEC-2020-53-01). The purpose and methods of the study were explained to all subjects, each of whom provided informed consent to participate.

The selection criteria for the experimental group were as follows: (1) age from 12–18 years; (2) full permanent dentition with second molar occlusion; (3) lack of contact between the lower incisors and the upper incisors or palate, and a vertical distance between the lower and upper incisal edge of at least 0.5 mm; (4) hyperdivergent facial type: SN-MP ≥ 40°; and (5) no missing teeth. The selection criteria for the control group were as follows: (1) age from 12 to 18 years; (2) full permanent dentition with second molar occlusion; (3) normal overbite: vertical distance between the lower and upper incisal edge of 1–4 mm; (4) normodivergent facial type: 30° < SN-MP < 40°; (5) no missing teeth; and (6) bilateral neutral molar relationship. The exclusion criteria for both groups were as follows: (1) previous orthodontic treatment or orthognathic surgery; (2) symptoms of temporomandibular joint disorder; (3) severe skeletal facial asymmetry; and (4) unilateral masticatory habit.

### EMG examination

**Fig. 1:**
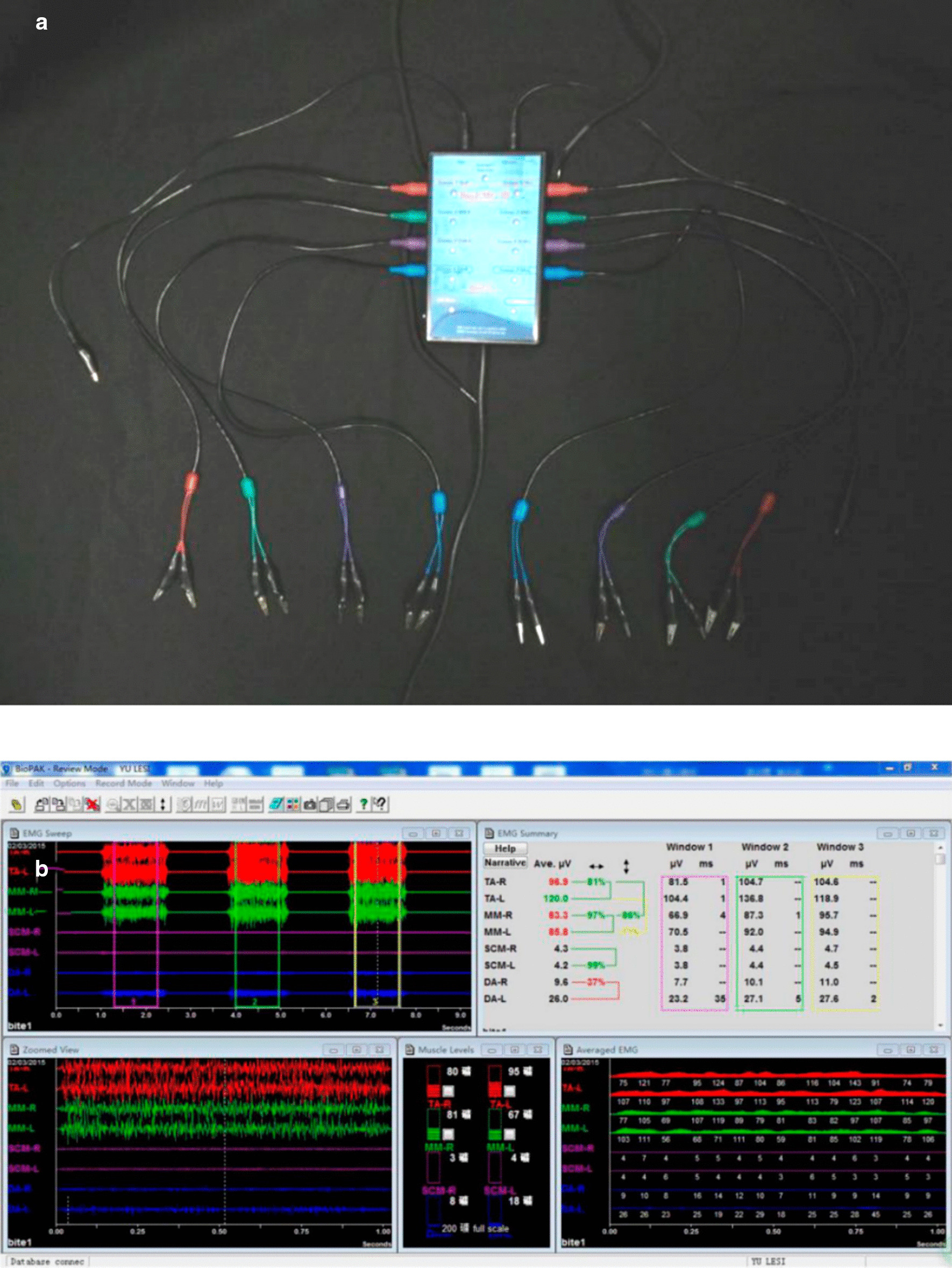
BioEMG, BioEMG electromyographic amplifier (**a**) and interface of recording and analysising of EMG (**b**)

**Fig. 2:**
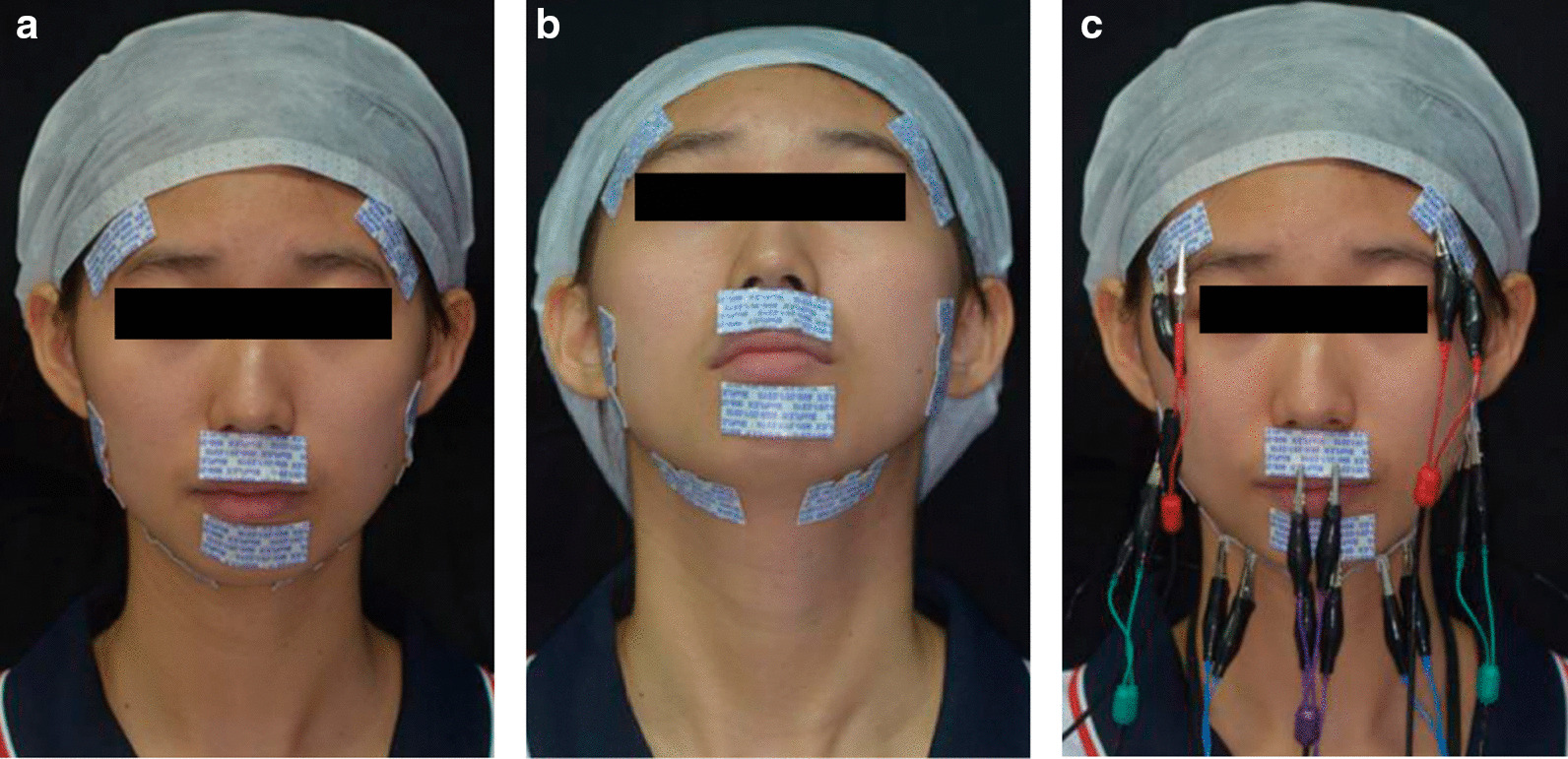
Differential active electrodes were placed along the main direction of the muscle fibers (**a**, **b**), electromyographic recordings with BioEMG electromyographic amplifier (**c**)

EMG recordings of the oromaxillofacial muscles were made using a BioEMG electromyographic amplifier (BioEMG, Bioresearch, Inc., Milwaukee, WI, USA) and BioPAC software (BioPAC Systems, Inc., Santa Barbara, CA, USA) (Fig. 1). The EMG examinations were conducted in the EMG laboratory of the orthodontic department of the Hospital of Stomatology, Sun Yat-sen University. Data were recorded with each subject seated in a dental chair; the head was supported with the Frankfort horizontal plane parallel to the ground. The position of differential active electrodes, determined by palpation and anatomical location, was parallel with the muscle fibres’ main direction, and skin impedance was reduced by wiping the skin with alcohol. In every case, a ground electrode was placed near the 7^th^ vertebra to assist the bipolar electrode configuration. In this way, EMGA associated with mastication, including the activity of anterior temporalis, masseter muscle, and anterior digastric, and EMGA associated with the closed mouth state, including the activity of upper orbicularis and mentalis muscle, were recorded (Fig. 2).

The EMG recordings were carried out under the following clinical conditions: mandibular rest (REST), maximum voluntary contraction in the intercuspal position (ICP), lip sealing (LIP), and swallowing (SWA). The protocols were as follows: REST: Subjects were asked to relax facial muscles for 5 min with the mandible in the rest position and the lips and teeth not in contact. ICP: The teeth were clenched in the ICP as tightly as possible for 2 s. LIP: Before testing, the lips were ensured to be moist and the mandible was in the rest position; then, the lips were slightly closed lips to achieve natural contact for 2 s while receiving instruction. SWA: First, 20 ml of water was held in the mouth and the facial muscles were relaxed for at least 5 s; then, the water was swallowed in one gulp while receiving instruction. EMGA under each condition was obtained three times, resting EMGA was measured for 5 s each time, EMGA under the other three conditions was assessed 2 s each time, with an interval of 1 min in order to prevent muscular fatigue. The mean values of EMGA of those muscles under each condition were calculated and recorded.

### Morphological characteristic evaluation

Before orthodontic treatment, the craniofacial morphological characteristics of all 36 subjects were measured using lateral cephalograms. Radiographs were obtained when the upper and lower dentition were in the centric occlusion position and the Frankfort horizontal plane was parallel to the ground. The radiographs were digitally traced using Quick Ceph Studio software (Quick Ceph Systems, San Diego, CA, USA).

The following cephalometric measurements were traced and measured to express different anatomic relationships: anteroposterior jaw relationships: SNA, SNB, and ANB; protrusion of incisors: Mx1-SN, Md1-MP, and angle of the axis of the upper and lower central incisors; mandibular plane inclination: FMA and GoGn-SN; facial height: N-Me, ANS-Me, Go-S, and Go-Ar; positional relations between the upper and lower incisors: overbite and overjet; and alveolar height: U1-PP, L1-MP, U6-PP, and L6-MP.

### OMT

**Fig. 3:**
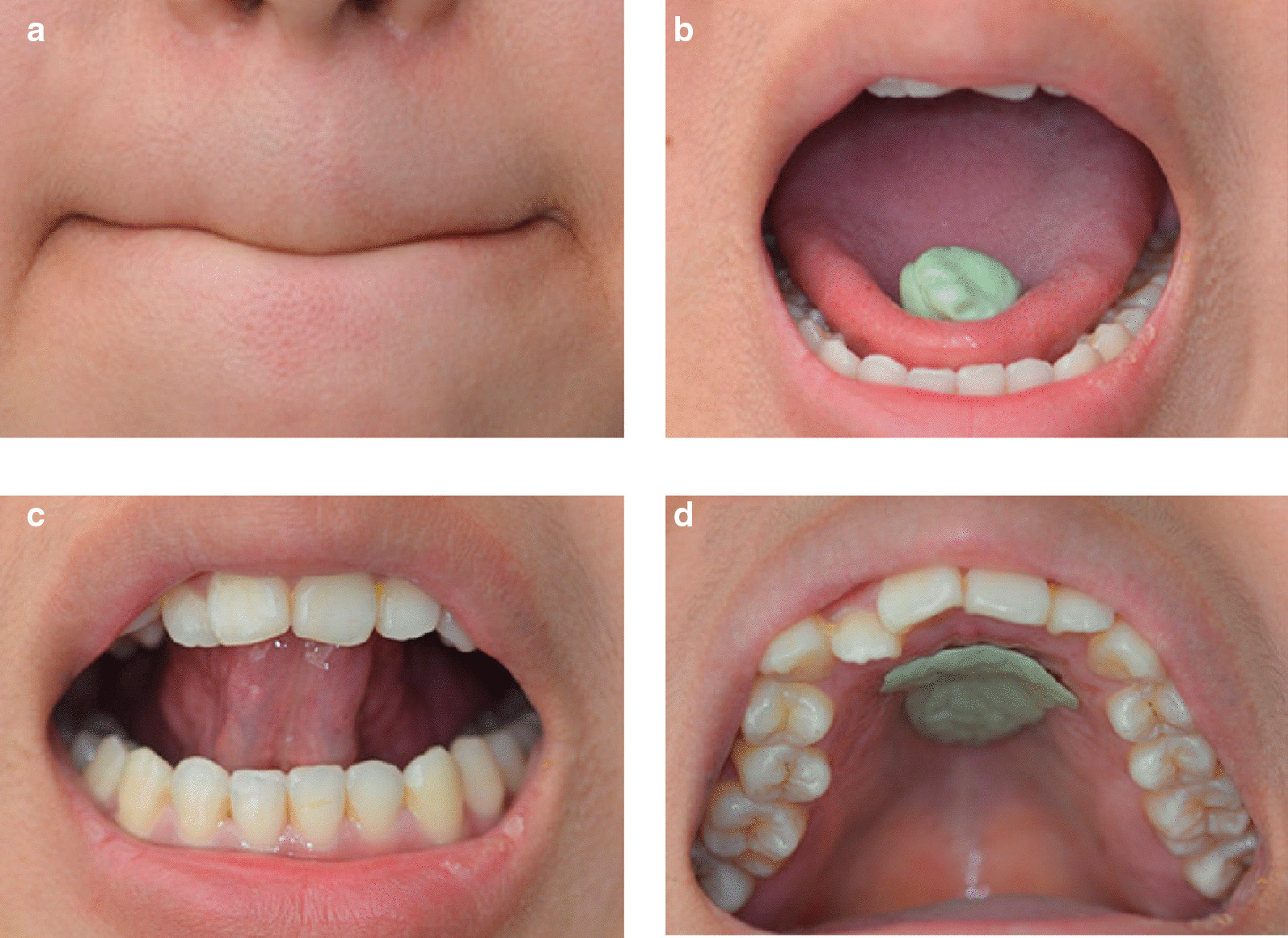
OMT, OMT of perioral muscles (**a**), OMT of masticatory muscles and tongue: including chewing gum vigorously with posterior teeth and shaping the softened chewing gum into a ball on the front of the tongue (**b**), lifting the tip of the tongue and sticking the gum to the hard palate (**c**), pressing the tongue against the hard palate and flattening the gum (**d**)

According to the myofunctional and morphological characteristics of the SAOB subjects, the perioral muscles, tongue and masticatory muscles were specially trained to achieve a better myofunctional status. The protocol was as follows: The perioral muscles were strengthened by pressing the lips tightly together for nasal breathing for at least 0.5 h a day (Fig. 3a). The masticatory muscles and tongue were trained for 2 h a day by chewing gum vigorously with posterior teeth and shaping the softened chewing gum into a ball on the front of the tongue, lifting the tip of the tongue and sticking the gum to the hard palate, pressing the tongue against the hard palate and flattening the gum, swallowing saliva while simultaneously pressing the tongue against the gum, and repeating the above steps (Fig. 3b).

The OMT regimen lasted for 3 months and subjects received follow-up contact by telephone every 2 weeks and paid a return visit every month. Each subject received an EMGA record before and after the 3-month OMT treatment.

### Statistical analysis

Wilcoxon signed rank tests and t-tests were performed to evaluate myofunctional and morphological differences between the normal and SAOB subjects. In order to select representative items, factor analysis was performed for 18 cephalometric measurements and 20 EMG measurements; then, the main factors from each group were extracted. Furthermore, we investigated the correlations between muscle activity and morphological characteristics by performing Pearson or Spearman correlation analysis of the main factors from among the cephalometric and EMG measurements. The OMS of SAOB subjects before and after receiving OMT was compared using a Wilcoxon signed rank test or t-test to investigate the effect of OMT. Wilcoxon signed rank tests were used for data with a skewed distribution, and t-tests were used for data with a normal distribution; α = 0.05. Data are presented as the median (25%, 75%) or mean ± standard deviation. A *P* value less than 0.05 was considered statistically significant. All tests were conducted using SPSS 19 software (SPSS, Chicago, IL, USA).

## Results

### OMS features and morphological characteristics of SAOB

At rest, the activity of anterior temporalis and mentalis muscle significantly increased in SAOB subjects compared to normal subjects, but the activity of anterior temporalis and masseter muscle decreased in the ICP. Furthermore, upper orbicularis activity and mentalis muscle activity significantly increased during lip sealing and swallowing, indicating that the OMS features of SAOB mainly include aberrant swallowing patterns and weak masticatory muscles (Table 1).

**Table 1:** Comparison of EMG activity between SAOB and normal subjects in each clinical conditions

	TA			MM			DA			UO			ME		
	SAOB μV	Normal μV	*P*	SAOB μV	Normal μV	*P*	SAOB μV	Normal μV	*P*	SAOB μV	Normal μV	*P*	SAOB μV	Normal μV	*P*
REST	1.53 (1.18, 2.23)^a^	1.03 (0.89, 1.21)^a^	< 0.001	0.93 (0.79, 1.36)^a^	0.80 (0.73, 1.05)^a^	0.134	0.95 (0.79, 1.34)	0.90 (0.88, 1.41)	0.424	1.10 (1.00, 1.45)^a^	0.90 (0.78, 1.30)^a^	0.470	4.60 (1.71, 7.20)^a^	1.38 (0.80, 2.16)^a^	< 0.001
ICP	37.3 (33.3, 59.2)^a^	86.2 (73.4, 103)^a^	< 0.001	27.5 ± 11.9^b^	83.1 ± 26.9^b^	< 0.001	4.68 ± 1.95	12.6 ± 7.42	< 0.001	2.30 (1.90, 2.83)^a^	2.50 (1.93, 3.20)^a^	0.481	4.93 (3.24, 7.35)^a^	5.45 (4.25, 7.20)^a^	0.104
LIP	1.93 (1.37, 2.61)^a^	1.23 (0.89, 1.86)^a^	0.024	1.38 (0.94, 1.96)^a^	0.90 (0.83, 1.21)^a^	0.05	1.48 (1.19, 3.25)	1.30 (0.98, 1.71)	0.118	14.0 (12.3, 18.0)^a^	4.30 (2.40, 7.05)^a^	< 0.001	17.7 ± 8.41^b^	6.80 ± 2.30^b^	< 0.001
SWA	2.58 (1.40, 3.25)^a^	2.53 (1.48, 4.91)^a^	0.913	3.20 (2.13, 5.15)^a^	3.20 (2.44, 4.91)^a^	0.719	7.85 (6.44, 10.6)	9.58 (7.84, 12.5)	0.214	25.4 (23.4, 26.9)^a^	9.95 (4.48, 11.7)^a^	< 0.001	30.0 (18.2, 48.1)^a^	12.6 (9.23, 17.8)^a^	< 0.001

Wilcoxon signed rank test was used for skew distribution data, t-test was used for normal distribution data, α = 0.05

TA, temporal muscle; MM, masseter muscle; DA, anterior digastric; UO, upper orbicularis; ME, mentalis muscle; REST, mandibular rest; ICP, maximum voluntary contraction in the intercuspal position; LIP, lip sealing; SWA, swallowing

^a^Median (25%, 75%)

^b^Mean ± standard deviation

Morphological evaluation of SAOB subjects compared to normal subjects revealed significant (*P* < 0.05) increases in the FMA, GoGn-SN, ANS-Me, N-Me, L1-MP, U6-PP, and L6-MP and significant (*P* < 0.05) decreases in the angle of the axis of the upper and lower central incisors and overbite. These findings indicate that the morphological features of SAOB mainly reflect a greater anterior facial height and greater degree of incisor protrusion (Table 2).

**Table 2:** Comparison of morphology characteristics between SAOB and normal subjects

	SAOB	Normal subjects	*P*
SNA	81.8 ± 3.58^a^	82.4 ± 3.32	0.62
SNB	77.9 ± 4.59	79.5 ± 3.00	0.21
ANB	4.00 ± 2.21	2.85 ± 2.09	0.12
Mx1-SN	117 ± 8.21	114 ± 5.94	0.18
Md1-MP	94.5 ± 9.09	96.4 ± 5.73	0.47
Angle of axis of upper and lower central incisor	106 ± 12.0	114 ± 9.08	0.03
FMA	34.0 (27.9, 35.3)^b^	24.3 (22.6, 26.6)	0.001
GoGn-SN	43.3 ± 5.85	34.4 ± 4.78	< 0.001
N-Me	120 (114, 125)	112 (111, 115)	0.006
ANS-Me	67.7 (66.2, 73.9)	61.5 (59.8, 64.8)	< 0.001
Go-S	72.8 ± 6.78	74.8 ± 4.88	0.31
Go-Ar	46.1 (45.0, 48.1)	45.8 (42.3, 49.7)	0.50
Overbite	− 1.80 (− 3.55, − 1.18)	0.80 (0.13, 2.33)	< 0.001
Overjet	4.45 (2.40, 6.50)	4.20 (3.15, 6.30)	0.91
U1-PP	28.6 ± 2.38	27.5 ± 2.48	0.19
L1-MP	40.8 ± 3.58	38.3 ± 1.83	0.01
U6-PP	20.3 (18.7, 22.4)	18.4 (17.4, 20.5)	0.02
L6-MP	27.6 ± 3.54	25.2 ± 2.14	0.02

Wilcoxon signed rank test was used for skew distribution data, t-test was used for normal distribution data, α = 0.05

^a^Median (25%, 75%)

^b^Mean ± standard deviation

### Correlations between EMGA and morphological characteristics of SAOB

Factor analysis was carried for 18 cephalometric measurements, and 5 main factors were extracted (Table 3). In the same way, factor analysis was carried for 20 EMG measurements, including the EMGA of 5 kinds of muscles under 4 different conditions, and 6 main factors were extracted (Table 4). The correlation analysis between the morphological factors and EMGA factors showed that factor 1 and factor 5 from the cephalometric measurements and factor 1 from the EMGA measurements were positively correlated (Table 5). Factor 1 from the cephalometric measurements corresponds to vertical height, including the ANS-Me, N-Me, U1-PP, U6-PP, L1-MP, FMA, GoGn-SN, L6-MP, and overbite, while factor 5 corresponds to incisor protrusion, including the Mx1-SN and angle of the axis of the upper and lower central incisors (Table 3). Factor 1 from the EMGA measurements corresponds to the high EMGA of perioral muscles and low EMGA of muscles associated with mastication in the ICP (Table 4).

**Table 3:** Factor loading of morphology characteristics by varimax rotation

Measurement	Factor 1	Factor 2	Factor 3	Factor 4	Factor 5
SNA	0.002	**0.909**	− 0.077	0.234	0.087
SNB	− 0.111	**0.851**	− 0.291	− 0.262	0.187
ANB	0.209	− 0.095	0.400	**0.826**	− 0.200
Mx1-SN	0.089	0.163	− 0.078	0.025	**0.957**
Md1-MP	− 0.281	− 0.089	**0.858**	0.105	0.050
Angle of axis of upper and lower central incisor	− 0.158	0.372	− 0.453	− 0.271	**−** **0.700**
FMA	**0.538**	**−** **0.592**	− 0.242	0.349	0.240
GoGn-SN	**0.512**	− 0.743	− 0.126	0.319	0.090
N-Me	**0.927**	− 0.058	0.178	− 0.154	− 0.009
ANS-Me	**0.979**	− 0.089	0.022	0.037	0.113
Go-S	0.279	**0.657**	0.364	− 0.406	− 0.142
Go-Ar	0.330	**0.598**	0.345	− 0.393	− 0.275
Overbite	**−** **0.620**	0.441	− 0.150	0.009	− 0.358
Overjet	− 0.105	− 0.143	− 0.105	**0.683**	0.265
U1-PP	**0.766**	− 0.162	− 0.248	0.060	− 0.145
L1-MP	**0.702**	0.152	0.448	0.206	0.125
U6-PP	**0.862**	0.113	− 0.011	− 0.043	0.068
L6-MP	**0.526**	0.118	**0.743**	− 0.111	0.003

The standardized factor loadings of factor 1 to factor 5 are as follows: 0.51, 0.59, 0.74, 0.68, 0.70. Measurements with strong correlation were extracted and displayed in a bold font

**Table 4:** Factor loading of EMGA by varimax rotation

Measurement	Factor 1	Factor 2	Factor 3	Factor 4	Factor 5	Factor6
TA1	0.306	0.158	**0.759**	0.301	0.145	− 0.118
MM1	0.257	**0.776**	− 0.013	− 0.079	0.283	− 0.120
DA1	− 0.229	**0.534**	0.174	0.170	0.242	**−** **0.433**
UO1	0.170	0.401	0.236	0.137	0.072	**−** **0.475**
ME1	**0.553**	0.175	0.027	− 0.169	**0.675**	− 0.099
TA2	**−** **0.854**	0.076	− 0.079	− 0.020	0.154	0.346
MM2	**−** **0.797**	0.095	− 0.194	− 0.154	− 0.151	0.352
DA2	**−** **0.808**	0.133	0.048	− 0.050	0.153	0.113
UO2	− 0.101	− 0.030	0.036	0.082	0.085	**0.799**
ME2	− 0.093	− 0.060	− 0.020	0.041	**0.906**	0.073
TA3	0.116	0.150	**0.864**	0.124	− 0.091	− 0.030
MM3	0.062	0.813	0.111	− 0.056	− 0.197	− 0.029
DA3	0.226	**0.556**	0.471	0.333	− 0.176	0.201
UO3	**0.771**	0.215	0.305	0.008	− 0.034	0.144
ME3	**0.801**	0.300	− 0.015	0.034	0.175	0.067
TA4	0.023	− 0.184	0.201	**0.781**	0.073	− 0.160
MM4	0.075	0.097	0.164	**0.825**	− 0.059	0.105
DA4	0.007	0.392	**−** **0.544**	**0.592**	− 0.084	0.104
UO4	**0.829**	0.140	0.315	− 0.077	− 0.045	0.087
ME4	**0.751**	0.260	0.019	0.136	0.296	0.054

TA1, MM1, DA1, UO1, ME1: EMGA of each muscles in resting status, TA2, MM2, DA2, UO2, ME2: EMGA of each muscles in ICP, TA3, MM3, DA3, UO3, ME3: EMGA of each muscles during lip sealing, TA4, MM4, DA4, UO4, ME4: EMGA of each muscle during swallowing

The standardized factor loadings of factor 1 to factor 6 are as follows: 0.55, 0.53, 0.54, 0.59, 0.67, 0.43. Measurements with strong correlation were extracted and displayed in a bold font

TA, temporal muscle; MM, masseter muscle; DA, anterior digastric; UO, upper orbicularis; ME, mentalis muscle

**Table 5:** The correlation between morphology factor and EMGA factor

	EMGA Factor 1	EMGA Factor 2	EMGA Factor 3	EMGA Factor 4	EMGA Factor 5	EMGA Factor 6
Morphology Factor 1	0.458^**^	0.123	0.269	− 0.078	0.146	− 0.087
Morphology Factor 2	− 0.284	0.035	− 0.054	0.103	0.168	0.125
Morphology Factor 3	0.101	0.174	− 0.270	− 0.078	− 0.126	0.115
Morphology Factor 4	0.219	− 0.277	0.059	0.148	0.191	0.064
Morphology Factor 5	0.385*	− 0.009	− 0.087	− 0.224	0.054	− 0.014

Pearson correlation analysis was used for normal distribution data, spearman correlation analysis was used for skew distribution data, *r* > 0.3. Measurements with strong correlation were extracted and displayed in a bold font

*P < 0.05, **P < 0.01

Table 6 shows the results of a further correlation analysis of the corresponding measurements of the main factors. The activity of anterior temporalis, masseter muscle and anterior digastric in the ICP were negatively correlated with measurements of vertical height, such as the ANS-Me, FMA, GoGn-SN, and Mx1-SN, while positively correlated with measurements of incisor protrusion, such as the angle of the axis of the upper and lower central incisors. Mentalis muscle activity and upper orbicularis activity were positively correlated with vertical height and negatively correlated with incisor protrusion during sealing lip and swallowing. At rest, mentalis muscle activity showed a positive correlation with vertical height and a negative correlation with incisor protrusion. These results suggest that the craniofacial dysmorphology of SAOB is associated with the orofacial myofunctional disorder. EMGA associated with mastication in the ICP was negatively correlated with vertical height and positively correlated with incisor protrusion, while EMGA associated with the closed mouth state was positively correlated with vertical height and negatively correlated with incisor protrusion.

**Table 6:** The correlation between measurements of morphology factor 1 and 5 and measurements of EMGA factor 1

	TA2	MM2	DA2	ME1	ME3	ME4	UO3	UO4
ANS-Me	**−** 0.469**	**−** 0.507**	**−** 0.332*	0.487**	0.494**	0.561**	0.396*	0.455**
N-Me	− 0.308	**−** 0.359*	− 0.228	**−** 0.332*	0.445**	0.269	0.306	0.245
FMA	**−** 0.660**	**−** 0.536**	**−** 0.347*	0.248	0.284	0.430**	0.294	0.405*
GoGn-SN	**−** 0.586**	**−** 0.444**	**−** 0.365*	0.208	0.262	0.414*	0.271	0.420*
U1-PP	− 0.077	− 0.135	0.018	0.052	0.166	0.336*	0.027	0.201
U6-PP	− 0.212	− 0.292	− 0.126	0.374*	0.299	0.386*	0.099	0.348*
L1-MP	− 0.259	− 0.287	− 0.235	0.488**	0.357*	0.376*	0.368*	0.164
L6-MP	− 0.104	− 0.203	− 0.153	0.397*	0.291	0.299	0.263	0.174
Overbite	0.723**	0.762**	0.543**	**−** 0.613**	**−** 0.531**	**−** 0.607**	**−** 0.453**	**−** 0.657**
Mx1-SN	**−** 0.333*	**−** 0.393*	**−** 0.494*	0.549**	0.343*	0.303*	0.167	0.280
Angle of Axis of Upper and Lower Central Incisor	0.494**	0.475**	0.334*	**−** 0.377*	**−** 0.326*	**−** 0.478**	− 0.189	− 0.286

Pearson correlation analysis was used for normal distribution data, spearman correlation analysis was for skew distribution data, *r* > 0.3. Measurements with strong correlation were extracted and displayed in a bold font

TA, temporal muscle; MM, masseter muscle; DA, anterior digastric; UO, upper orbicularis; ME, mentalis muscle

*P < 0.05; **P < 0.01

### Influence of OMT on EMGA in SAOB

By comparing the measurements, it was found that mentalis muscle activity at rest, and the activity of anterior temporalis, masseter muscle and anterior digastric in the ICP increased significantly (*P* < 0.05) after OMT; however upper orbicularis activity and mentalis muscle activity decreased significantly (*P* < 0.05) after OMT, suggesting that OMT might contribute to normalization of the OMS by strengthening the muscles associated with mastication in the ICP and relaxing the perioral muscles during lip sealing (Table 7).

**Table 7:** Comparison of EMG activity of SAOB before and after receiving OMT

	TA				MM				DA			
	Before μV	After μV	T μV	*P*	Before μV	After μV	T μV	*P*	Before μV	After μV	T μV	*P*
REST	1.60 (1.25, 2.30)^a^	1.45 (1.20, 1.85)	− 0.32 ± 0.87^b^	0.147	0.95 (0.80, 1.40)	1.45 (1.20, 1.85)	0.41 ± 0.78	0.06	0.90 (0.75, 1.60)	1.00 (0.70, 1.30)	− 0.03 ± 0.27	0.950
ICP	37.2 (31.5, 58.8)^a^	62.1 (51.2, 84.7)	25.4 (7.20, 31.9)	0.001	26.3 ± 12.2^b^	60.6 ± 37.6	34.4 ± 30.2	**0.003**	4.49 ± 1.94	8.68 ± 3.88	4.19 ± 3.54	< 0.001
LIP	1.85 (1.30, 2.65)^a^	1.70 (1.15, 2.10)	− 0.41 ± 0.98 ^b^	0.132	1.40 (0.95, 1.95)	1.45 (0.90, 1.55)	− 0.10 (− 0.55, 0.15)	0.158	1.80 (1.30, 3.40)	1.35 (0.90, 1.55)	− 1.05 ± 1.36	0.005
SWA	2.45 (1.40, 5.05)^a^	3.00 (1.60, 6.40)	0.75 ± 3.63^b^	0.820	3.65 (2.15, 5.45)	3.40 (2.05, 5.15)	− 0.15 ± 2.78	1.000	8.70 (6.95, 11.05)	7.80 (5.85, 16.5)	− 0.60 (− 2.30, 1.10)	0.629

	UO				ME			
	Before μV	After μV	T μV	*P*	Before μV	After μV	T μV	*P*
REST	1.10 (1.00, 1.40)	1.10 (0.80, 1.50)	0.00 (− 0.30, 0.30)	0.916	5.20 (1.80, 7.10)	2.50 (1.65, 4.90)	− 0.80 (− 2.70, − 0.30)	0.002
ICP	2.30 (1.90, 2.90)	2.20 (1.80, 2.90)	− 0.20 (− 0.60, 1.00)	0.489	3.90 (3.05, 8.10)	6.15 (3.95, 12.1)	0.45 (− 1.30, 3.00)	0.233
LIP	15.5 (12.4, 18.7)	3.20 (1.80, 8.10)	− 10.4 ± 3.53	0.001	18.8 ± 8.51	9.86 ± 4.56	− 8.97 ± 6.49	0.008
SWA	23.7 (16.8, 26.5)	10.4 (6.90, 18.4)	− 10.1 ± 8.13	0.002	34.8 ± 18.8	20.6 ± 12.6	− 14.3 ± 8.76	< 0.001

Wilcoxon signed rank test was used for skew distribution data, t-test was used for normal distribution data, α = 0.05

TA, temporal muscle; MM, masseter muscle; DA, anterior digastric; UO, upper orbicularis; ME, mentalis muscle; REST, mandibular rest; ICP, maximum voluntary contraction in the intercuspal position; LIP, lip sealing; SWA, swallowing

^a^Median (25%, 75%)

^b^Mean ± standard deviation

## Discussion

A general agreement has been reached that orofacial myofunctional disorder has strong ties to malocclusion and the craniofacial dysmorphology of SAOB. Among various methods to estimate the neuromuscular status, EMG has become the most common method because of the higher sensitivity of the myoelectric amplitude than indicators of morphology and function in reflecting myofunctional changes. In this research, by comparing the EMGA of different muscles under four conditions between adolescents with SAOB or normal occlusion, we found that the OMS features of SAOB mainly include aberrant swallowing patterns and weak masticatory muscles. Furthermore, the morphological features of SAOB were found to mainly involve the anterior facial height and degree of incisor protrusion, and the correlation analysis revealed that the craniofacial dysmorphology of SAOB is associated with orofacial myofunctional disorder.

In this research, orofacial EMGA was classified into EMGA associated with mastication, including the activity of anterior temporalis, masseter muscle, and anterior digastric, and EMGA associated with the closed mouth state, including activity of upper orbicularis and mentalis muscle, since mastication and the closed mouth state are common and important states during orofacial function. Mastication is a complex functional movement integrating the jaw bones, masticatory muscles and soft tissues, such as the cheeks, lips and tongue. The temporal muscle, masseter muscle, and anterior digastric muscle play important roles in mastication by contracting or relaxing muscle fibres to lift or lower the mandible and close or open the mouth. Regarding perioral muscles, the upper orbicularis and mentalis muscle are essential for maintaining the closed mouth state at rest and during functions such as swallowing and lip sealing.

The ICP is the most important functional position during mastication. In this research, we observed that in the ICP, the activity of anterior temporalis, masseter muscle and anterior digastric were significantly lower in adolescents with SAOB, which is in agreement with the findings of previous studies [20–22]; additionally, there were significant negative correlations between vertical morphology factors with the activity of all three of these muscles in the ICP. It is widely accepted that a large proportion of subjects with SAOB suffer from mouth breathing [6, 8]. Breathing is the primary requirement, so the oral cavity needs to make room for air circulation, and then food often cannot be chewed fully. As masticatory force and nasal breathing can both influence the three-dimensional development of the jaws during growth, the low mastication efficiency and lack of chewing stimulation in the molar area can lead to an increase in the occlusal height and eventually the formation of hyperdivergent facial pattern [20]. In this research, subjects in the experimental group had already formed steep occlusal plane angles. The reason for this finding may lie in that some morphological features of SAOB, such as a higher gonial angle and greater maxillary height, can shift the position of the load application point posteriorly, which leads to an increase in the loading moment arm and eventually results in weakening of the mechanical force of the stomatognathic system. Furthermore, the cross-sectional area and muscular force of the masseter muscle and temporal muscle tend to be smaller in those with a hyperdivergent facial type [23], which results in lower EMGA.

The clinical rest position is also called the mandibular postural position, in which the mandible stretches masticatory muscle fibres by the effect of gravity to maintain the mandibular position. Subjects with SAOB usually show a well-developed and clockwise-rotated mandibular body, which imposes a greater stretching force through the masticatory muscles. In the present study, the anterior temporalis activity in the experimental group was markedly higher than that in the control group in the resting posture, while the difference in masseter muscle activity was not significant; Cha [24] reported similar findings in a previous study. This result might be explained by the different functions of the temporal muscle and masseter muscle in the resting position and ICP potentially providing different stimuli to the corresponding neuromuscular spindles.

In turns of perioral muscles, harmful habits, such as mouth breathing and tongue-thrust swallowing, during development can disrupt the balance between perioral muscles and other muscles, resulting in malocclusion. In this research, the EMGA in SAOB patients was significantly higher at rest and during lip pressing and swallowing and was positively correlated with the anterior facial height but negatively correlated with incisor protrusion. The reason for this finding may be that the high prevalence of harmful oral habits, such as mouth breathing, tongue-thrust swallowing and thumb sucking, during development results in lip incompetence as a common phenomenon in subjects with SAOB [25–27]. In the normal stomatognathic system, the muscular forces of perioral muscles and the tongue maintain a balance to achieve passive lip contact and eventually a closed oral environment, which means that achieving a normal lip sealing state does not require greater contraction of the perioral muscle fibres. Tomiyama [28] found that subjects with incompetent lips showed higher EMGA in the clinical rest position or during chewing when they were told to close their mouth. Gamboa [27] found that the EMGA of perioral muscles was significantly higher in subjects without competent lips during swallowing. Thus, subjects with SAOB tend to exert greater perioral muscular effort due to the requirement of lip sealing during functional activities. Furthermore, incompetent lips contribute to harmful oral habits, such as chronic mouth breathing and tongue thrusting, which could be risk factors for open bite and are associated with the adaptive change of the hyperdivergent facial pattern during development [29, 30].

On this basis, the OMS feature of the SAOB facial pattern was found to mainly include passive lip contact with extra perioral muscle contraction, aberrant swallowing patterns, and weak masticatory muscles. According to these features, well-directed OMT was given to adolescents with SAOB, and the OMT phase of the intervention lasted 3 months. After the treatment, the EMGA of masticatory muscles was significantly increased, while that of perioral muscles was decreased, indicating that the OMT contributed to an improvement in the orofacial myofunctional disorder. As development of the masticatory system is influenced by functional needs, gum chewing exercise was designed to improve masticatory function in this research. Previous studies have shown that chewing exercise contributes to improvements in masticatory disturbances and deficiencies by influencing the functional capacity and increasing the strength of the masticatory muscles [31–33], which is consistent with our results. Furthermore, according to the high prevalence of harmful oral habits, such as mouth breathing and tongue-thrust swallowing, among subjects with SAOB and the relationship of these habits with orofacial myofunctional disorder, lip-pressing training and tongue training were designed to correct the harmful oral habits and abnormal tongue forces by establishing patterns of nasal breathing and physiological swallowing. Former studies have demonstrated that OMT can significantly change the tongue posture by establishing patterns of nasal breathing and physiological swallowing [30, 34]. Our results support the efficacy of OMT in altering the OMS. Combined with the findings discussed above, OMT is effective in correcting harmful habits and harmonizing OMS abnormalities, indicating that OMT as one of adjuvant orthodontic therapies is beneficial to the treatment of SAOB patients. For patients in the growth and development stage, in whom the malocclusion or craniofacial deformities has not yet formed, it is important to raise awareness of harmful oral habits and provide early intervention with well-directed OMT to help rebalance the OMS for facial bone growth. For patients in whom malocclusion or craniofacial deformities has already developed, combining OMT with conventional orthodontic treatment may help reduce the difficulty of treatment by harmonizing abnormal OMS. However, there have been few studies on the effects of OMT on the improvement of craniofacial morphology in SAOB patients. Further research is needed to evaluate the morphological changes of SAOB patients after OMT and confirm the long-term effects of OMT as an adjunct to conventional orthodontic treatment. It is worth noting that genetic factors should also be taken into account.

## Conclusion

In summary, the OMS features of SAOB mainly include aberrant swallowing patterns and weak masticatory muscles, while the morphological features mainly reflect a greater anterior facial height and greater degree of incisor protrusion, demonstrating that the craniofacial dysmorphology of SAOB is associated with orofacial myofunctional disorder. Furthermore, OMT contributes to OMS harmonization by normalizing abnormal EMGA, indicating that taking OMT as one of adjuvant orthodontic therapies is beneficial to the development and treatment of SAOB.

## Data Availability

Data used and/or analysed during the current study are available from the corresponding author on reasonable request.

## Abbreviations

SAOB: Skeletal anterior open bite
OMS: Orofacial myofunctional status
OMT: Orofacial myofunctional therapy
EMGA: Electromyographic activity
TAA: Anterior temporalis activity
MMA: Masseter muscle activity
DAA: Anterior digastric activity
UOA: Upper orbicularis activity
MEA: Mentalis muscle activity
AOB: Anterior open bite
EMG: Electromyography
TA: Temporal muscle
MM: Masseter muscle
OMT: Orofacial myofunctional therapy
REST: Mandibular rest
ICP: Maximum voluntary contraction in the intercuspal position
LIP: Lip sealing
SWA: Swallowing
DA: Anterior digastric
UO: Upper orbicularis
ME: Mentalis muscle
